# High-density Lipoproteins and Apolipoprotein A-I: Potential New Players in the Prevention and Treatment of Lung Disease

**DOI:** 10.3389/fphar.2016.00323

**Published:** 2016-09-21

**Authors:** Elizabeth M. Gordon, Debbie M. Figueroa, Amisha V. Barochia, Xianglan Yao, Stewart J. Levine

**Affiliations:** Laboratory of Asthma and Lung Inflammation, Cardiovascular and Pulmonary Branch, National Heart, Lung, and Blood Institute, National Institutes of Health, Bethesda, MDUSA

**Keywords:** high-density lipoprotein, Apolipoprotein A-I, lung diseases, apolipoprotein mimetic peptides

## Abstract

Apolipoprotein A-I (apoA-I) and high-density lipoproteins (HDL) mediate reverse cholesterol transport out of cells. Furthermore, HDL has additional protective functions, which include anti-oxidative, anti-inflammatory, anti-apoptotic, and vasoprotective effects. In contrast, HDL can become dysfunctional with a reduction in both cholesterol eﬄux and anti-inflammatory properties in the setting of disease or the acute phase response. These paradigms are increasingly being recognized to be active in the pulmonary system, where apoA-I and HDL have protective effects in normal lung health, as well as in a variety of disease states, including acute lung injury (ALI), asthma, chronic obstructive pulmonary disease, lung cancer, pulmonary arterial hypertension, pulmonary fibrosis, and viral pneumonia. Similar to observations in cardiovascular disease, however, HDL may become dysfunctional and contribute to disease pathogenesis in respiratory disorders. Furthermore, synthetic apoA-I mimetic peptides have been shown to have protective effects in animal models of ALI, asthma, pulmonary hypertension, and influenza pneumonia. These findings provide evidence to support the concept that apoA-I mimetic peptides might be developed into a new treatment that can either prevent or attenuate the manifestations of lung diseases, such as asthma. Thus, the lung is positioned to take a page from the cardiovascular disease playbook and utilize the protective properties of HDL and apoA-I as a novel therapeutic approach.

High-density lipoprotein particles (HDL) and its major protein, apolipoprotein A-I (apoA-I), play key roles in attenuating the risk of atherosclerotic cardiovascular disease (Navab et al., 2011; Kingwell et al., 2014; Rader and Hovingh, 2014; Rosenson et al., 2016). A key mechanism by which HDL decreases atherosclerosis is by mediating reverse cholesterol eﬄux out of cells. ApoA-I, which is primarily produced in the liver and small intestine, can form nascent, discoidal HDL particles by complexing with unesterified cholesterol and phospholipids that have been eﬄuxed via the ATP-binding cassette subfamily A member 1 (ABCA1) transporter (Kingwell et al., 2014). Following esterification and transport of cholesterol to the particle core by lecithin-cholesterol acyltransferase, spheroidal HDL particles can eﬄux additional cholesterol and phospholipids from cells via ABCG1 and scavenger receptor class B member 1 (SRB1). In addition, HDL has anti-oxidative, anti-inflammatory, anti-apoptotic, and vasoprotective properties (Navab et al., 2011; Rosenson et al., 2016). An anti-inflammatory function of HDL is its ability to reduce the cholesterol content of immune cells by decreasing receptor localization and signaling within lipid rafts (Catapano et al., 2014). However, in the setting of disease or the acute-phase response, HDL can become dysfunctional, with diminished cholesterol eﬄux and anti-inflammatory properties (Kingwell et al., 2014; Rosenson et al., 2016). It is increasingly recognized that both HDL (**Figure 1**) and apoA-I (**Figure 2**) similarly, play important roles in modulating normal lung health and disease. Thus, paradigms that have long been applied to atherosclerotic cardiovascular disease may also be relevant for pulmonary disorders, including acute lung injury (ALI), asthma, chronic obstructive pulmonary disease, lung cancer, pulmonary fibrosis, pulmonary hypertension, and viral pneumonia.

**FIGURE 1:**
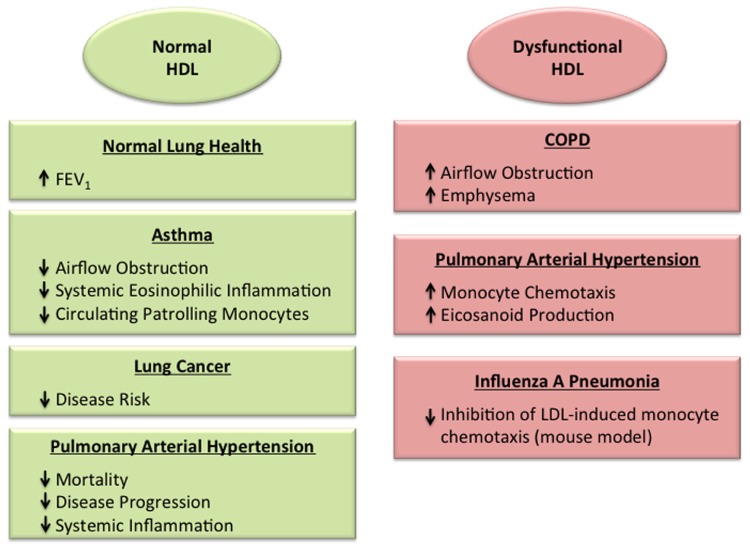
**Roles of normal and dysfunctional high-density lipoproteins (HDL) in the lung.** HDL has been associated with protective effects in the normal lung (Cirillo et al., 2002), as well as in the setting of asthma (Barochia et al., 2015, 2016; Rastogi et al., 2015) and lung cancer (Kucharska-Newton et al., 2008; Chi et al., 2014; Chandler et al., 2016), which may reflect its anti-inflammatory and anti-oxidant properties. In contrast, dysfunctional HDL particles may promote disease severity in the setting of chronic obstructive pulmonary disease (Burkart et al., 2014) and viral pneumonia (Van Lenten et al., 2001). HDL particles in pulmonary arterial hypertension have been found to have either protective effects (Heresi et al., 2010; Sharma et al., 2014), or dysfunctional, pro-inflammatory properties (Ross et al., 2015).

**FIGURE 2:**
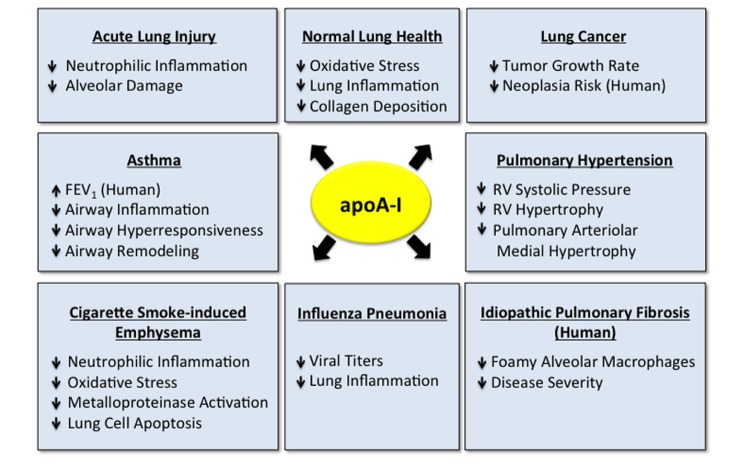
**Apolipoprotein A-I (apoA-I) has multiple protective functions in the normal and diseased lung.** Apolipoprotein A-I has multiple protective and beneficial functions in the normal lung (Wang et al., 2010) and in a variety of pulmonary disorders, including acute lung injury (ALI) (Yan et al., 2006; Jiao and Wu, 2008; Li et al., 2008; Van Linthout et al., 2011; Kwon et al., 2012; Madenspacher et al., 2012; Sharifov et al., 2013), asthma (Nandedkar et al., 2011; Dai et al., 2012; Yao et al., 2011; Park et al., 2013), cigarette smoke-induced emphysema (Kim et al., 2016), influenza pneumonia (Van Lenten et al., 2002, 2004), lung cancer (Zamanian-Daryoush et al., 2013), pulmonary hypertension (Sharma et al., 2014) and idiopathic pulmonary fibrosis (IPF) (Kim et al., 2010; Lee et al., 2013). Data addressing the role of apoA-I in lung disease have primarily been generated in experimental animal models, although several studies have suggested that apoA-I may be associated with decreased disease severity in subjects with asthma (Barochia et al., 2015, 2016) and IPF (Kim et al., 2010), which are indicated.

## Normal Lung Development and Lung Lipid Homeostasis

Levels of mRNA encoding apoA-I, apoA-II, and apoH, are expressed at higher levels in the murine fetal lung than the adult lung, which suggests that pulmonary apolipoprotein production modulates lung maturation via lipid metabolism and transport during embryonic development (Provost et al., 2009). Apolipoprotein A-I and apoA-II are primarily expressed by capillary-like structures during gestational days 15.5–18.5 (Cote et al., 2011). In the adult lung, apoA-I is expressed by alveolar epithelial cells and alveolar macrophages, while the ABCA1 transporter is expressed by type I and type II alveolar epithelial cells, alveolar macrophages, pulmonary vascular endothelial cells (PVECs), and airway smooth muscle cells (Bortnick et al., 2003; Bates et al., 2008; Delvecchio et al., 2008; Kim et al., 2010; Dai et al., 2012, 2014; Park et al., 2013).

The apoA-I/ABCA1 pathway maintains normal lipid homeostasis in the lung by mediating the release of lipids from type I alveolar epithelial cells to apoA-I (Bates et al., 2008). Similarly, ABCA1 expression by type II alveolar epithelial cells facilitates the removal of cholesterol mass and reduces basal rates of surfactant secretion (Bortnick et al., 2003). The important role of ABCA1 in maintaining normal lipid homeostasis in the lung is further highlighted by the finding that alveolar macrophages and type II alveolar epithelial cells from *Abca1*-deficient mice are enriched with cholesterol (McNeish et al., 2000; Bates et al., 2005). Furthermore, *Abca1*-deficient mice have abnormal lung morphology characterized by alveolar proteinosis, as well as hypertrophy and hyperplasia of type II alveolar epithelial cells.

Studies utilizing *Apoa1*-deficient mice have shown an important role for apoA-I in attenuating basal levels of inflammation and oxidative stress in the lung. For example, HDL from *Apoa1*-deficient mice oxidizes at a faster rate than HDL from wild-type mice (Wang et al., 2010). Furthermore, *Apoa1*-deficient mice display increased systemic oxidative stress, lung inflammation, and collagen deposition. Basal levels of airway hyperresponsiveness (AHR) are also increased in *Apoa1*-deficient mice, while pulmonary artery vasodilatation is impaired. Thus, apoA-I plays an important role in maintaining normal lung health.

## Acute Lung Injury (ALI) and Acute Respiratory Distress Syndrome (ARDS)

Acute respiratory distress syndrome describes a clinical entity of severe lung dysfunction that reflects an acute inflammatory lung injury secondary to a variety of causative factors, such as bacterial infection (Bellani et al., 2016). A beneficial property of apoA-I is its ability to directly bind and neutralize lipopolysaccharide (LPS), which is derived from the cell walls of gram-negative bacteria, as well as lipoteichoic acid (LTA), which is a cell wall component of gram-positive bacteria (Emancipator et al., 1992; Ma et al., 2004; Jiao and Wu, 2008; Henning et al., 2011; Van Linthout et al., 2011; Sharifov et al., 2013). The domains that mediate LPS binding are located in the carboxy-terminus of apoA-I (Henning et al., 2011). Apolipoprotein A-I can also associate with the lipopolysaccharide-binding protein, which binds and neutralizes LPS (Wurfel et al., 1994).

Both murine and human studies have provided evidence for a protective function of apoA-I in ALI and ARDS. *Apoa1*-deficient mice have increased neutrophil recruitment to the lung following inhalation of LPS or direct instillation of CXCL1, which can be attenuated by administration of the L-4F apoA-I mimetic peptide by a mechanism involving inhibition of CXCR2-mediated neutrophil chemotaxis (Madenspacher et al., 2012). Similarly, administration of apoA-I mimetic peptides, human apoA-I, or over-expression of human apoA-I via a recombinant adenoviral vector, protected rodents from developing neutrophilic airway inflammation and ALI in models of LPS- or LTA-mediated systemic inflammation (Yan et al., 2006; Jiao and Wu, 2008; Li et al., 2008; Van Linthout et al., 2011; Kwon et al., 2012; Sharifov et al., 2013). Treatment of LPS-stimulated human blood with the L-4F apoA-I mimetic peptide has suppressed endotoxin activity and IL-6 secretion, as well as preserved paraoxonase function (Sharifov et al., 2013). The L-4F apoA-I mimetic peptide also inhibited cellular activation and superoxide formation when human neutrophils were exposed to serum obtained from subjects with ARDS. Administration of human HDL to mice has also attenuated LPS-induced ALI (Xiao et al., 2008).

Human translational studies have suggested a correlation between the *APOA1* gene and the risk of sepsis-associated ALI. Carriage of the *APOA1* -75 AA genotype in the *APOA1* promoter, which has reduced promoter activity as compared to the major G allele, has been associated with a higher risk of ALI following cardiopulmonary bypass surgery (Smith et al., 1992; Tu et al., 2013). The rs11216153 SNP in the *APOA1* gene has also been associated with ALI risk in subjects with sepsis, with the GG genotype and G allele being more common among ALI patients than controls (Hao and He, 2014). However, since this SNP is located in a non-coding region of the *APOA1* gene, it is unclear if the association is with the *APOA1* gene itself or variants from nearby genes.

## Asthma

Murine studies have demonstrated a protective role for an apoA-I/ABCA1 pathway in the pathogenesis of asthma. *Apoa1*-deficient mice that were sensitized and challenged with ovalbumin developed augmented neutrophilic airway inflammation that was primarily mediated by the increased production of granulocyte colony stimulating factor (G-CSF) by alveolar macrophages (AMϕs) and PVECs, as well as by the increased expression of IL-17A, TNF-α, CXCL5, and VCAM-1 (Dai et al., 2012). Furthermore, G-CSF can increase the number of tissue neutrophils by attenuating apoptosis, which prolongs neutrophil survival in the lung (Matute-Bello et al., 1997). Similarly, transgenic mice that expressed human ABCA1 under the control of the Tie2 promoter in both PVECs and AMϕs had decreased ovalbumin-induced neutrophilic airway inflammation that was in part mediated by the reduced expression of G-CSF (Dai et al., 2014).

Murine studies have further demonstrated that apoA-I mimetic peptides can suppress allergen-mediated airway inflammation. Administration of the 5A apoA-I mimetic peptide inhibited allergen-mediated increases in neutrophilic airway inflammation in *Apoa1*-deficient mice (Dai et al., 2012). Similarly, administration of the 5A apoA-I mimetic peptide to wild-type mice attenuated the development of allergen-induced airway inflammation and AHR, and also reduced airway remodeling responses, including mucous cell metaplasia and collagen gene expression (Yao et al., 2011). Likewise, administration of the D-4F apoA-I mimetic peptide suppressed oxidative stress, eosinophilic airway inflammation, AHR, and total immunoglobulin E levels in allergen-challenged wild-type mice (Nandedkar et al., 2011). Administration of human apoA-I to allergen-challenged wild-type mice similarly, suppressed airway inflammation, AHR, dendritic cell migration to the lung, and reduced the release of IL-25, IL-33, and TSLP (thymic stromal lymphopoietin) by airway epithelial cells (Park et al., 2013). In addition, apoA-I administration promoted the recovery of disrupted airway epithelial tight junction proteins and increased the pro-resolution lipid mediator, lipoxin A4, in both mice and humans. Human translational studies have also supported a role for apoA-I in asthma. For example, apoA-I levels in bronchoalveolar lavage fluid (BALF) from mild-to-moderate asthmatics are five-fold lower than in BALF from normal control subjects (Park et al., 2013). Similarly, apoA-I levels are reduced in BALF from ovalbumin-challenged wild-type mice as compared to control mice that were sensitized and challenged with saline (Dai et al., 2012).

Clinical studies have shown that higher systemic levels of HDL and apoA-I are associated with less severe airflow obstruction in both healthy individuals and asthmatics. For example, in an analysis of subjects without respiratory disease who participated in the Third National Health and Nutrition Survey, both HDL and apoA-I were positively associated with FEV_1_ (Cirillo et al., 2002). Similarly, both HDL and apoA-I were positively correlated with FEV_1_ in a cohort of atopic asthmatics at the National Heart, Lung, and Blood Institute (Barochia et al., 2015). Furthermore, the positive association with FEV_1_ in atopic asthmatics was mediated by the subset of large HDL_NMR_ particles, as quantified by nuclear magnetic resonance (NMR) spectroscopy. Higher circulating levels of HDL have also been correlated with lower blood eosinophil counts in asthma, which is a biomarker of type 2 inflammation (Barochia et al., 2016). The negative association between serum HDL and absolute blood eosinophil counts was again mediated by the subset of large HDL_NMR_ particles. Of note, large HDL_NMR_ particles have also been associated with a lower risk of incident cardiovascular disease and type 2 diabetes in women (Mora et al., 2009, 2010). Another correlation between HDL and inflammation was identified in obese, adolescent asthmatics who had an inverse association with the number of patrolling monocytes, which is an innate immune cell population that can be rapidly recruited to the lung during inflammation (Rastogi et al., 2015). Collectively, these studies suggest that apoA-I and HDL may modulate both airflow obstruction and airway inflammation in asthma.

## Chronic Obstructive Pulmonary Disease (COPD)

Chronic obstructive pulmonary disease is caused by inhalation of noxious particles or gases, such as cigarette smoke, with resultant chronic inflammation and airflow limitation that is not fully reversible (Celli et al., 2015). ApoA-I and HDL have context-dependent effects in COPD. For example, apoA-I has a protective role in preventing cigarette-smoke induced emphysema. In a murine model of cigarette smoke-induced lung disease, transgenic mice that conditionally over-expressed the human *APOA1* gene in alveolar epithelial cells were protected from developing emphysema (Kim et al., 2016). In particular, apoA-I over-expression attenuated cigarette smoke-induced increases in lung inflammation, oxidative stress, metalloproteinase activation, and lung cell apoptosis by a mechanism that involved the reduced translocation of Fas to lipid rafts, which decreased the formation of death-inducing signaling complexes and caspase-8 activation. Cigarette smoke exposure also decreased the amount of apoA-I in the lungs of wild-type mice, which suggests that the loss of its protective function may contribute to emphysema pathogenesis (Kim et al., 2016). Similarly, lung tissue from patients with moderate emphysema contained reduced amounts of apoA-I as compared to lung tissue from non-smoking subjects. Furthermore, the amount of apoA-I was reduced in induced sputum samples of COPD patients as compared to healthy smokers (Nicholas et al., 2010). These studies suggest that the reduction in lung apoA-I levels may contribute to lung disease in COPD.

High-density lipoproteins may also have a therapeutic role in emphysema caused by alpha-1-antitrypsin deficiency. Alpha-1-antitrypsin is a component of HDL that confers anti-protease properties that inhibit leukocyte elastase (Ortiz-Munoz et al., 2009). Intravenous administration of HDL enriched with alpha-1-antitrypsin to cigarette smoke-exposed mice prevented the development of pulmonary emphysema and also reduced the numbers of BALF neutrophils and macrophages, as well as BALF levels of IL-6, TNF-α (tumor necrosis factor), and MCP-1 (monocyte chemoattractant protein-1) (Moreno et al., 2014). BALF fibronectin degradation and matrix metalloproteinase (MMP-2 and MMP-9) activity were also reduced. The protective effects of intravenous administration of HDL complexed with alpha-1-antitrypsin were superior to those of HDL or alpha-1-antitrypsin alone, which suggested that combining HDL with alpha-1-antitrypsin might be more effective than the current strategy of alpha-1-antitrypsin augmentation therapy.

In contrast to the ability of apoA-I to attenuate the induction of cigarette smoke-induced emphysema in murine models, HDL has been associated with increased disease severity in human subjects with emphysema. In the MESA (Multi-Ethnic Study of Atherosclerosis) COPD study, higher plasma HDL levels were associated with a lower FEV_1_/FVC ratio, which indicated more severe airflow obstruction (Burkart et al., 2014). Furthermore, higher HDL levels were associated with greater percent emphysema on chest computed tomography scans. This suggests that HDL may not be protective in the setting of established emphysema, but instead may have dysfunctional properties (Navab et al., 2011; Kingwell et al., 2014; Rader and Hovingh, 2014; Rosenson et al., 2016).

## Lung Cancer

Potential anti-tumorigenic effects of apoA-I have been identified using murine neoplasia models that showed a dose-dependent inhibition of Lewis lung tumor growth rates with increasing apoA-I levels (Zamanian-Daryoush et al., 2013). Potential anti-neoplastic effects of apoA-I included inhibition of angiogenesis and MMP-9 activity, accumulation of myeloid-derived suppressor cells in tumor beds, and expression of the anti-apoptotic protein, survivin. In addition apoA-I, was found to promote the anti-neoplastic effects of tumor-associated CD11b^+^ macrophages.

The prospective Atherosclerosis Risk in Communities Study found an inverse association between serum HDL-C levels and incident lung cancer over a 13 years period in former smokers, but not in current smokers (Kucharska-Newton et al., 2008). Similarly, an inverse association between low serum HDL-C and neoplasia was seen with other cancers, which suggests a common mechanism by which low HDL-C levels may promote an environment that is permissive to neoplasia. Another study of non-small cell lung cancer (NSCLC) subjects found that serum HDL-C levels were significantly decreased as compared to normal controls (Chi et al., 2014). Furthermore, NSCLC subjects with low levels of HDL-C had significantly reduced cumulative 5-year survival rates as compared to individuals with normal HDL-C levels. The Women’s Health Study prospectively associated incident cancer rates and cancer mortality with circulating lipid biomarkers in health professionals (Chandler et al., 2016). This study found that higher circulating levels of apoA-I and HDL were inversely associated with the risk of both total cancer and lung cancer. Collectively, these data support an anti-neoplastic role for apoA-I and HDL in lung cancer.

## Pulmonary Hypertension

Apolipoprotein A-I and HDL may also have context-dependent effects in pulmonary hypertension. For example, administration of the 4F apoA-I mimetic peptide rescued preexisting pulmonary hypertension in rodent models by increasing the expression of the microRNA, mmu-miR-193, via the retinoid X receptor α (Sharma et al., 2014). Induction of mmu-miR-193 inhibited the expression of insulin-like growth factor-1 receptor and lipoxygenases (e.g., ALOX5, ALOX12, and ALOX15), which suppressed pulmonary artery smooth muscle cell proliferation.

Human translational studies have shown that HDL may be associated with disease severity and outcome in pulmonary arterial hypertension (PAH). For example, a study of PAH subjects at the Cleveland Clinic found significantly lower serum levels of HDL-C as compared to control subjects (Heresi et al., 2010). Furthermore, when subjects were stratified by HDL-C levels, those with HDL-C greater than 35 mg/dl had no mortality and a longer time to clinical worsening over a period of 1.5 years, which was independent of underlying cardiovascular risk factors, insulin resistance, or PAH disease severity. Furthermore, PAH subjects with HDL-C levels that exceeded 35 mg/dl had less systemic inflammation, as indicated by lower levels of serum C-reactive protein, TNF-α, IL-17A, CCL2, and ICAM-1. In contrast, another study of subjects with “idiopathic” PAH (IPAH) or PAH “associated” with underlying etiologies (APAH) found that HDL had been converted to a dysfunctional, pro-inflammatory particle which increased the chemotaxis of peripheral blood monocytes as compared to normal HDL (Ross et al., 2015). *Ex vivo* treatment with the 4F apoA-I mimetic peptide significantly improved, but did not completely attenuate, the dysfunctional pro-inflammatory HDL phenotype. In addition, HDL fractions from IPAH and APAH subjects had increased levels of multiple eicosanoids. Therefore, additional studies will be needed to further define the roles of HDL in PAH.

## Pulmonary Fibrosis

Idiopathic pulmonary fibrosis (IPF) is a disease of chronic, progressive fibrosing interstitial pneumonia that occurs primarily in older adults (Raghu et al., 2015). Reduced amounts of apoA-I have been found in BALF from subjects with IPF as compared to normal controls (Kim et al., 2010). Furthermore, apoA-I levels in BALF from IPF patients were inversely correlated with both clinical severity scores and the percentage of foamy macrophages. The role of apoA-I in pulmonary fibrotic disease was subsequently assessed in murine models, where intranasal administration of human apoA-I protein suppressed the number of BALF inflammatory cells, histologic evidence of inflammatory cell infiltration, and lung collagen deposition. Furthermore, apoA-I levels were reduced in BALF from bleomycin-challenged mice. Similarly, transgenic mice that over-expressed human apoA-I in alveolar epithelial cells were protected from silica-induced lung inflammation and fibrotic nodule formulation by a mechanism that might involve increased expression of lipoxin A4 (Lee et al., 2013). Collectively, these data suggest that apoA-I has a protective effect in IPF.

## Viral Pneumonia

Apolipoprotein A-I and HDL also modulate the pathogenesis of influenza A infection. HDL isolated from mice infected with influenza A is dysfunctional with reduced anti-inflammatory and anti-oxidant properties that, in part, reflected decreased activity of paraoxonase and platelet-activating factor acetylhydrolase, as well as increased ceruloplasmin, which is a potent oxidant (Van Lenten et al., 2001). In contrast, apolipoprotein J levels in HDL were increased, which might promote cholesterol eﬄux from macrophages. Administration of the D-4F apoA-I mimetic peptide to mice infected with influenza A attenuated lung inflammation, suppressed IL-6 production, and prevented macrophage trafficking into arteries (Van Lenten et al., 2002). The D-4F apoA-I mimetic peptide also had antiviral activity that reduced influenza titers by more than 50%. Similarly, the D-4F apoA-I mimetic peptide inhibited the production of oxidized phospholipids, IFN-α/β, and IL-6 by an alveolar epithelial cell line and also suppressed viral titers and caspase activity (Van Lenten et al., 2004). Collectively, these findings suggest that apoA-I mimetic peptides may have therapeutic potential for the treatment of influenza A pneumonia.

## Conclusion

An increasing body of evidence supports the concept that HDL, and particularly apoA-I, modulate the pathogenesis and severity of lung disease. This may involve both the systemic effects of HDL in the circulation, as well as the local production of apoA-I in the lung. Although normal HDL has multiple protective effects, HDL may become dysfunctional in the setting of lung disease and no longer attenuate disease manifestations. Furthermore, data from murine models has suggested that small apoA-I mimetic peptides, which are based upon the α-helical structure of apoA-I, may be developed into a novel therapy for lung disease. In particular, administration of 5A and D-4F apoA-I mimetic peptides has protective effects in animal models of ALI, asthma, pulmonary hypertension, and influenza pneumonia (Yao et al., 2011; Van Lenten et al., 2002; Dai et al., 2012; Kwon et al., 2012; Madenspacher et al., 2012; Sharifov et al., 2013; Sharma et al., 2014). Furthermore, the D-4F and L-4F apoA-I mimetic peptides have already been evaluated in human subjects with cardiovascular disease and have been well-tolerated (Dunbar et al., 2007; Bloedon et al., 2008; Watson et al., 2011). This provides evidence to support the concept that inhalational administration of apoA-I mimetic peptides to provide site-directed delivery to the lung may attenuate the manifestations of pulmonary disease. Consistent with this concept, our laboratory is currently working toward advancing an inhaled formulation of the 5A apoA-I mimetic peptide to clinical trials that will assess its safety and role for the treatment of asthma. Therefore, the lung may benefit from taking a page from the cardiovascular disease playbook and apply the protective properties of HDL and apoA-I as a novel therapeutic approach for pulmonary disease.

## Author Contributions

All authors listed, have made substantial, direct and intellectual contribution to the work, and approved it for publication.

## Conflict of Interest Statement

The authors declare that the research was conducted in the absence of any commercial or financial relationships that could be construed as a potential conflict of interest.
